# Monocular Visual Measurement System Uncertainty Analysis and One-Step End–End Estimation Upgrade

**DOI:** 10.3390/s25237179

**Published:** 2025-11-24

**Authors:** Kuai Zhou, Wenmin Chu, Peng Zhao

**Affiliations:** 1 School of Aeronautical Engineering, Nanjing University of Industry Technology, Nanjing 210023, China; 2 College of Mechanical and Electrical Engineering, Nanjing University of Aeronautics and Astronautics, Nanjing 210016, China

**Keywords:** monocular vision, aircraft assembly, uncertainty analysis, one-step end-to-end, pose estimation

## Abstract

Monocular visual measurement and vision-guided robotics technology find extensive application in modern automated manufacturing, particularly in aerospace assembly. However, during assembly pose measurement and guidance, the propagation and accumulation of multi-source errors—including those from visual measurement, hand–eye calibration, and robot calibration—impact final assembly accuracy. To address this issue, this study first proposes an uncertainty analysis method for monocular visual measurement systems in assembly pose, encompassing the determination of uncertainty propagation paths and input uncertainty values. Building on this foundation, the system’s uncertainty is analyzed. Inspired by the uncertainty analysis results, this study further proposes a direct one-step solution to a series of problems in robot calibration and hand–eye calibration using a nonlinear mapping estimation method. Through experiments and discussion, a high-performance, one-step, end-to-end pose estimation convolutional neural network (OECNN) is constructed. The OECNN achieves direct mapping from the pose variation of the target object to the drive volume variation of the positioner. The uncertainty analysis conducted in this study yields a series of conclusions that are significant for further enhancing the precision of assembly pose estimation. The proposed uncertainty analysis methodology may also serve as a reference for uncertainty analysis in complex systems. Experimental validation demonstrates that the proposed one-step end-to-end pose estimation method exhibits high accuracy. It can be applied to automated assembly tasks involving various vision-guided robots, including those with typical configurations, and it is particularly suitable for high-precision assembly scenarios, such as aircraft assembly.

## 1. Introduction

Modern industry uses industrial robots to automate production tasks. Industrial robots were initially widely used in the automotive industry [1] to handle tasks with high precision and high repeatability, including welding, painting, gluing, and assembly. In addition to the automotive industry, automated manufacturing based on industrial robots has also been applied to aerospace manufacturing [2,3], pharmaceutical manufacturing [4,5], and metal processing [6,7]. In the past, the automation of industrial robots was mainly achieved through offline programming, relying on the repeatability of robots. This method is relatively rigid and requires a lot of time and effort for programming and debugging. It is especially unsuitable for the aerospace manufacturing field, which has extremely high manufacturing precision requirements [8,9]. To solve this problem, measurement-assisted manufacturing [10] has emerged in the aerospace manufacturing field. Through various measurement systems, including laser trackers [11], lidar [12], iGPS [13], and visual measurement [14], industrial robots are guided to move and flexibly complete various manufacturing tasks. The importance of measurement systems to overall manufacturing precision is self-evident. Among the above-mentioned measurement systems, monocular visual measurement has the advantages of small size, high measurement accuracy, and strong environmental adaptability [15]. It has been widely used in the field of aerospace manufacturing, especially for high-precision assembly tasks in narrow spaces. However, during the process of monocular visual measurement-guided assembly, various errors and uncertainties are transmitted, affecting overall assembly accuracy [16]. Therefore, evaluating the uncertainty of the entire assembly pose measurement system is very important for error compensation to improve overall assembly accuracy, and it can also provide guidance for subsequent related research.

The monocular visual measurement system for assembly mainly consists of an industrial robot and an industrial camera, which is installed at the end of the industrial robot. The uncertainty determination of the assembly pose measurement system can be achieved through theoretical analysis, simulation experiments, and statistical methods [17]. There are many studies on uncertainty analysis of visual measurement systems, especially stereo vision measurement systems. Leo [18] proposed an uncertainty evaluation method based on the uncertainty propagation law. Reu [19] studied the influence of calibration uncertainty on system measurement uncertainty and used the Monte Carlo method to evaluate the calibration uncertainty of stereo vision earlier, verifying the effectiveness of the Monte Carlo method in the uncertainty evaluation of complex systems. Zhu [20] estimated the error of three-dimensional reconstruction based on numerical simulation and static experiments, improving the convenience of measurement. Isa [21] developed a program for modeling, measuring, and correcting system errors of stereo vision systems based on experiments and statistics and then used neural networks for regression. Cui [22] proposed an uncertainty weighted pose measurement method, which used the covariance matrix to determine the uncertainty of feature points, and integrated it into the projection matrix of stereo vision pose estimation. Jiang [23] used the Monte Carlo method to analyze the uncertainty of the combined vision measurement system based on tracking. Chen [24] used the GUM method (Guide to the Expression of Uncertainty in Measurement) to analyze the uncertainty of the dynamic stereo vision measurement system and obtained an analytical solution. Zhao [25] analyzed the uncertainty of the line laser scanning measurement system and compared the advantages and disadvantages of the GUM method and the Monte Carlo method. GUM is suitable for models that are relatively simple and have good linearity. When the measurement model is linear or has low nonlinearity and the inputs are independent, the GUM method is accurate and efficient. For example, the calibration models for some traditional instruments are usually quite standard. The Monte Carlo method, on the other hand, is suitable for highly nonlinear or complex models. If error propagation involves multiple stages and the model is relatively complex, MCM can more accurately reflect the propagation of uncertainty. MCM effectively overcomes the limitations of the GUM method when dealing with specific complex models, providing evaluation results that better reflect actual probability distributions. Ngeljaratan [26] analyzed the uncertainty of stereo photogrammetry. Guo [27] used the error decoupling method to model the error of the stereo vision system. The above studies are limited to the scope of visual measurement and did not extend the uncertainty analysis to the level of assembly posture. There are few studies on the analysis of assembly posture uncertainty. Deng [28] analyzed the uncertainty of docking of large aircraft components. Gai [29] evaluated the uncertainty of large surface measurement systems in aircraft assembly. Unfortunately, the measurement equipment used in the assembly systems studied by the two was a laser tracker, and no analysis was conducted on the monocular visual measurement system.

On the other hand, in the digital docking system of large aircraft components, three-coordinate CNC positioners are usually used as motion actuators to adjust the pose of large aircraft components [30]. The positioner is an electromechanical automatic device that moves in the *X*, *Y*, and *Z* directions according to a predetermined trajectory during the pose adjustment process [31,32]. Multiple positioners are connected to the bracket that fixes the large aircraft components to form a parallel robot [33]. The assembly accuracy of vision-guided robots, including parallel robots, is affected by visual measurement accuracy, hand–eye calibration accuracy, and robot calibration accuracy [16]. Simply improving the visual measurement accuracy while ignoring the hand–eye calibration accuracy and robot calibration accuracy cannot effectively improve the assembly accuracy to meet the requirements of engineering use. According to different implementation principles, the existing hand–eye calibration methods can be divided into traditional hand–eye calibration methods and neural-network-based hand–eye calibration methods. Traditional hand–eye calibration methods can be further divided into two categories. The first category aims to first calculate the rotation matrix in the hand–eye relationship matrix and then calculate the translation vector. This method is called the separation method. The second category aims to calculate the rotation matrix and the translation vector at the same time. It is called the synchronization method. Representatives of the separation method include the rotation-matrix-based method proposed by Shiu [34], Tsai [35], and others and the Lie group algebra-based method proposed by Park [36]. The synchronization method is represented by the analytical solution proposed by Lu [37], Danielidis [38], and others and the numerical solution based on the Newton gradient method proposed by Gwak [39]. In recent years, with the vigorous development of deep learning technology, scholars have gradually tried to use deep learning methods to solve the problem of robot hand–eye calibration. Hua [40] proposed a hand–eye calibration method based on optimized neural networks, which directly maps the image information captured by the camera to the posture information of the end of the robot, realizes hand–eye calibration on a two-dimensional plane, and improves the final calibration accuracy, but at the cost of increasing the time required for calibration. Zhou [16] went a step further and proposed a posture estimation method based on convolutional neural networks, which replaced the traditional hand–eye calibration process and implicitly embedded the hand–eye calibration parameters in the neural network, effectively improving the accuracy of hand–eye calibration.

The general steps of robot calibration include kinematic parameter modeling, pose measurement, parameter identification, and error compensation [41]. Chen [42] analyzed the application of an asymmetric driven 3-PPPS parallel robot in the process of aircraft wing adjustment and used a kinematic calibration method to improve the docking accuracy of the terminal posture. Deng [28] proposed a new relative pose estimation model considering the uncertainty of critical point anisotropy measurement and solved it using a particle swarm optimization algorithm to improve the calibration accuracy. Wang [43] proposed a fast calibration method based on a sequential quadratic programming optimization algorithm for the calibration of a movable CNC positioner coordinate frame in an aircraft digital assembly system, ensuring positioner positioning accuracy up to 0.05 mm, thereby ensuring the joint positioning accuracy of the CNC positioner group. Chu [44] proposed a motion parameter calibration method based on closed-loop feedback of force for a redundantly driven parallel mechanism and optimized the solution using the RANSAC method. However, all of these robot calibration methods require the use of external measurement equipment, such as laser trackers or force sensors, and the uncertainty of the measurement equipment itself limits the further improvement of robot calibration accuracy.

In summary, no researchers have yet analyzed the uncertainty of monocular visual measurement systems for assembly poses. This has led to a lack of theoretical guidance for research on core technologies related to monocular visual measurement, hindering further improvements in assembly pose accuracy. Furthermore, the aforementioned hand–eye and robot calibration methods each have their own limitations, which significantly impact final assembly accuracy. To address these two issues, this study proposes a Monte Carlo simulation-based uncertainty assessment method for assembly pose monocular visual measurement systems. This method begins with pixel uncertainty in the industrial camera coordinate system and then analyzes visual measurement uncertainty and, finally, assembly pose uncertainty. Inspired by the uncertainty analysis results, this study further proposes a one-step, end-to-end pose estimation method based on a convolutional neural network. This method implicitly incorporates the hand–eye relationship matrix and robot calibration parameters into the trained neural network model. During use, the target object’s pose variation in the camera coordinate system is input, and the positioner’s drive volume variation is directly output, driving the positioner for pose adjustment during assembly. Experimental results demonstrate that this method effectively improves final assembly accuracy.

The contributions of this paper are as follows:

- An assembly pose visual measurement system is established to determine the system uncertainty transmission path. The system uncertainty is analyzed using the Monte Carlo method, and a series of conclusions are drawn.
- Inspired by the uncertainty analysis results, a new one-step end-to-end pose estimation method is proposed, which covers and replaces the robot calibration and hand–eye calibration processes.
- A data acquisition method for training is designed. By experimenting with various classic neural network architectures, a new one-dimensional convolutional neural network model for end-to-end pose estimation is constructed.

The remainder of this paper is organized as follows. Section 2 introduces the assembly pose visual measurement system, which is abstracted into a vision-guided robot model as the basis for subsequent research. Section 3 conducts an uncertainty analysis based on the Monte Carlo method, determines the system uncertainty transmission route and uncertainty analysis implementation plan, and obtains the numerical value of the input uncertainty through repeated experiments. The Monte Carlo method is then used to analyze the uncertainty of the system and obtain relevant conclusions. Section 4 focuses on the one-step end-to-end estimation method, including the positioner calibration principle, the advantages of one-step end-to-end estimation, and the implementation process. Section 5 details the implementation details of the proposed convolutional neural network, including the acquisition and preprocessing of training data, the determination of the basic neural network architecture, and the discussion of the optimal network architecture and hyperparameters. Section 6 validates the accuracy and effectiveness of the proposed method through a series of experiments, while also demonstrating that this approach can be applied to vision-guided robot configurations beyond typical setups.

## 2. Assembly Pose Visual Measurement Model

**Figure 1:**
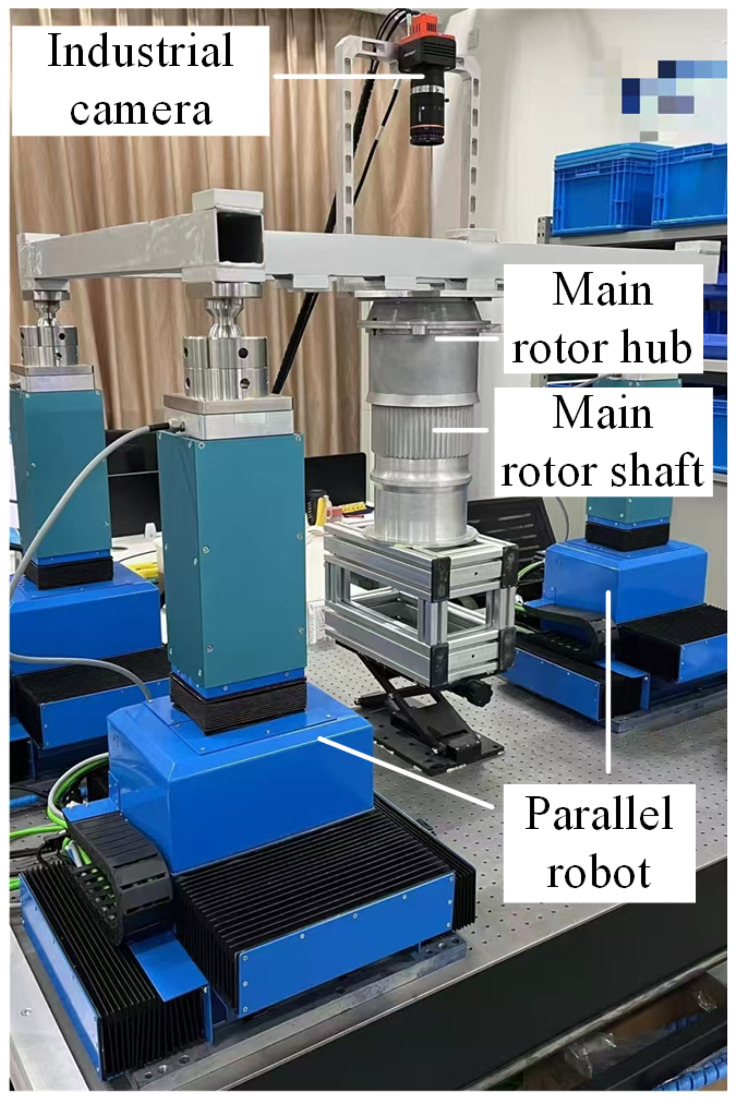
Aerospace structure hole–shaft assembly system.

The assembly pose visual measurement model covers the entire process from image acquisition with an industrial camera to computational assembly pose, involving a camera imaging model, a camera calibration model, and a robot hand–eye calibration model. To facilitate the study of aerospace structural hole–shaft assembly problems in a laboratory setting, this paper first summarizes the real-world aerospace structural hole–shaft assembly system into a general abstract model. Figure 1 shows a true-to-scale simulation of a helicopter lift system assembly, consisting of the main rotor hub (main hub) and main rotor shaft. The main rotor shaft is fixed to a base coordinate system, and the main hub is fixed to the end of a 6-DOF parallel robot. An industrial camera mounted on the end of the robot visually guides the robot to control the main hub’s pose and complete assembly.

In the aerospace structure hole–shaft assembly system shown in Figure 1, the pose of the main hub/main rotor shaft is obtained through visual measurement, which is *H_OC_*. The pose relationship between the camera and the robot end is obtained through robot hand–eye calibration, which is *H_CH_*. The pose of the robot end in the base coordinate system is *H_HB_*. In order to complete the assembly task, the robot needs to determine the pose *H_OB_* of the target in the base coordinate system, which is

(1) $$H_{OB}=H_{HB}\times H_{CH}\times H_{OC}$$

**Figure 2:**
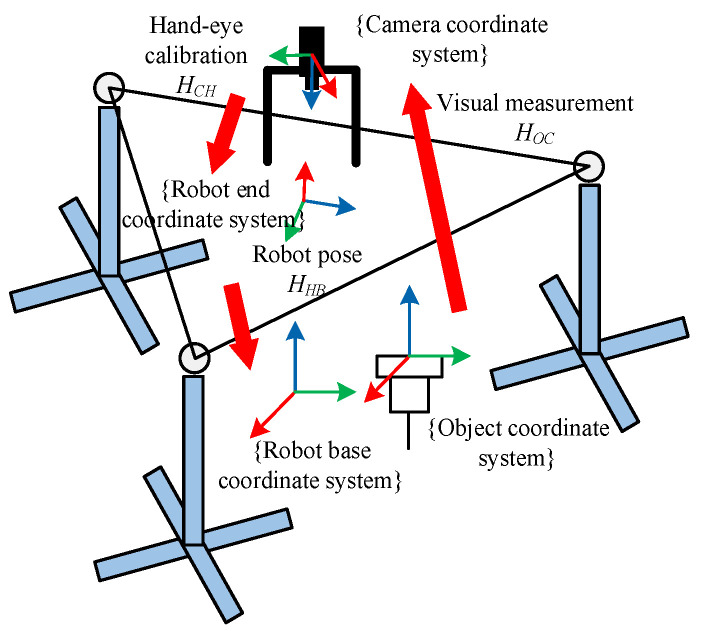
Vision-guided robot model.

According to Equation (1), the aerospace structure hole–shaft assembly system in Figure 1 can be abstracted into a vision-guided robot model, as shown in Figure 2.

The assembly accuracy of a vision-guided robot is determined by three factors: visual measurement accuracy, hand–eye calibration accuracy, and robot positioning accuracy. Robot positioning accuracy involves robot calibration. To facilitate the subsequent uncertainty analysis, the object’s position in the robot’s end coordinate system is defined as the assembly pose *H_OH_*, which is

(2) $$H_{OH}=H_{CH}\times H_{OC}$$

After obtaining the assembly pose *H_OH_*, it is only necessary to determine the robot’s current pose *H_HB_* to complete the assembly, that is,

(3) $$H_{OB}=H_{HB}\times H_{OH}$$

## 3. Uncertainty Analysis Based on Monte Carlo Method

### 3.1. System Uncertainty Transfer

**Figure 3:**
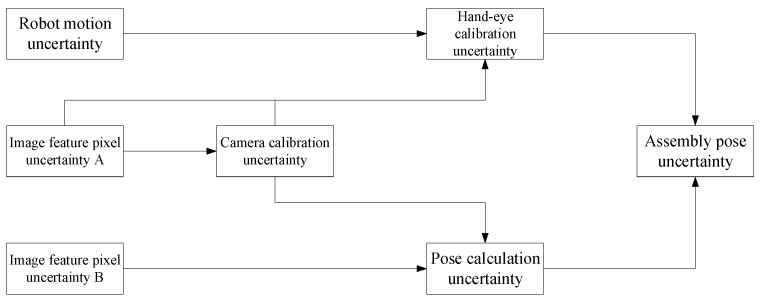
Uncertainty transfer process of assembly pose combination system.

In an assembly pose combination system, the final assembly pose is not only dependent on visual measurement but also influenced by hand–eye calibration. Uncertainty is a measure of system accuracy and can be used as an evaluation method for system precision and stability. It is particularly important for analyzing uncertainty in complex systems. Therefore, this article analyzes the uncertainty of an assembly pose combination system to provide guidance for improving assembly pose accuracy and, ultimately, for assembly applications. Figure 3 illustrates the process of transferring input uncertainty to the final assembly pose uncertainty in the assembly pose combination system.

In Figure 3, image feature pixel uncertainty A represents the pixel uncertainty of the camera’s extraction of corner features from the checkerboard calibration plate during camera calibration or hand–eye calibration, while image feature pixel uncertainty B represents the pixel uncertainty of the camera’s extraction of relevant features from the target image during pose calculation. Assembly pose uncertainty is determined by both pose calculation uncertainty and hand–eye calibration uncertainty. The uncertainties of the various components in Figure 3 arise from interactions, including the following.

1. Pose calculation uncertainty: The raw data of the pose calculation process are the target image data. The pose of the target in the camera coordinate system is output by combining the relevant features in the target image with the camera intrinsic parameter data obtained through calibration. Therefore, the sources of pose calculation uncertainty are image feature pixel uncertainty B and camera calibration uncertainty.
2. Hand–eye calibration uncertainty: The Horaud hand–eye calibration method [45] is used. The raw data required for hand–eye calibration are several calibration plate images captured by industrial cameras and the corresponding robot end pose. First, the corner point features in the calibration plate image are extracted, and the pose of the calibration plate in the camera coordinate system is calculated based on the camera intrinsic parameter. Then, the hand–eye calibration is completed by combining the corresponding robot end pose, and the hand–eye relationship matrix is output. Therefore, the sources of hand–eye calibration uncertainty are image feature pixel uncertainty A, camera calibration uncertainty, and robot motion uncertainty.
3. Camera calibration uncertainty: The raw data required for camera calibration are several calibration plate images captured by industrial cameras. The corner point features in the calibration plate images are extracted, and the camera internal parameters are output. Therefore, the source of camera calibration uncertainty is the image feature pixel uncertainty A.

### 3.2. Uncertainty Analysis Implementation Plan

The International Standardization Organization (ISO) stipulates that for complex systems whose uncertainty cannot be accurately estimated, the uncertainty can be analyzed using the Monte Carlo method [46]. This paper uses the Monte Carlo method to analyze the uncertainty of the assembly posture combination system. This paper uses the Monte Carlo method to analyze the uncertainty of the assembly posture combination system. The specific implementation process is as follows:

1. Obtain image pixel uncertainty A, image pixel uncertainty B, and robot motion uncertainty as input uncertainties.
2. Calculate camera calibration uncertainty: Add the image pixel uncertainty A obtained in (1) to the image features used for camera calibration to generate calibration two-dimensional feature points and corresponding three-dimensional space points. Then, repeatedly run the camera calibration program and calculate the standard deviation of the output parameters, which is recorded as the camera calibration uncertainty.
3. Calculate pose calculation uncertainty: Add the image pixel uncertainty B obtained in (1) to a certain image feature used for pose calculation, add the camera uncertainty obtained in (2) to the camera intrinsic parameters, and then repeatedly run the pose calculation program and calculate the standard deviation of the output parameters, which is recorded as the pose calculation uncertainty.
4. Calculate the uncertainty of hand–eye calibration: Add the image pixel uncertainty A obtained in (1) to the image features used for hand–eye calibration, add the camera uncertainty obtained in (2) to the camera intrinsic parameters, and obtain the position of the calibration plate in the camera coordinate system. Then, add the robot motion uncertainty obtained in (1) to the robot position used for hand–eye calibration. Then, repeatedly run the hand–eye calibration program and calculate the standard deviation of the output parameters, which is recorded as the uncertainty of hand–eye calibration.
5. Calculate the uncertainty of assembly pose: Add the pose calculation uncertainty obtained in (3) to the pose of the target in the camera coordinate system. Add the hand–eye calibration uncertainty obtained in (4) to the hand–eye relationship matrix. Then, repeatedly run the assembly pose calculation program and calculate the standard deviation of the output parameters, which is recorded as the uncertainty of assembly pose.

### 3.3. Determination of Input Uncertainty

First, it is necessary to obtain the input uncertainties of the assembly pose combination system—image pixel uncertainty A, image pixel uncertainty B, and robot motion uncertainty. Evaluating the system’s input uncertainty is crucial for the subsequent assessment of assembly pose uncertainty. Numerous factors influence image pixel uncertainty and robot motion uncertainty. For example, image pixel uncertainty includes illumination variations, image noise, and image feature detection algorithms. These factors are difficult to accurately model under varying environments. Therefore, this paper employs a repeated experimentation method to obtain image pixel uncertainty A and image pixel uncertainty B. Robot motion accuracy is directly used as the robot motion uncertainty.

Regarding the image pixel uncertainty A, the parameters of the industrial camera model used in the experiment are shown in Table 1. The features collected were checkerboard corner features. During the acquisition process, the camera frame rate was set to the highest possible rate to minimize the impact of environmental changes. Image acquisition was repeated 500 times, and the coordinate distribution of the same corner feature was recorded. The standard deviation in the u and v directions was calculated as the uncertainty of corner feature extraction, i.e., the image pixel uncertainty A, as shown in Table 2.

The image pixel uncertainty B, in conjunction with the pose calculation method, affects the pose calculation uncertainty. Because this section focuses on the uncertainty transfer process within the entire assembly pose visual measurement system, for the sake of operability, the pose of the checkerboard calibration plate in the camera coordinate system is directly calculated. The pose calculation method uses the PnP algorithm. The corresponding image pixel uncertainty B is the image pixel uncertainty A, i.e., the uncertainty of checkerboard corner feature extraction.

For the robot motion uncertainty, this article takes the positioning accuracy of the robot used as its motion uncertainty. The robot position positioning accuracy is 0.03 mm, and the attitude positioning accuracy is 5 × 10^−5^ rad. Therefore, the robot position motion uncertainty is set to 0.03 mm, and the attitude motion uncertainty is set to 5 × 10^−5^ rad.

### 3.4. System Uncertainty Analysis

**Figure 4:**
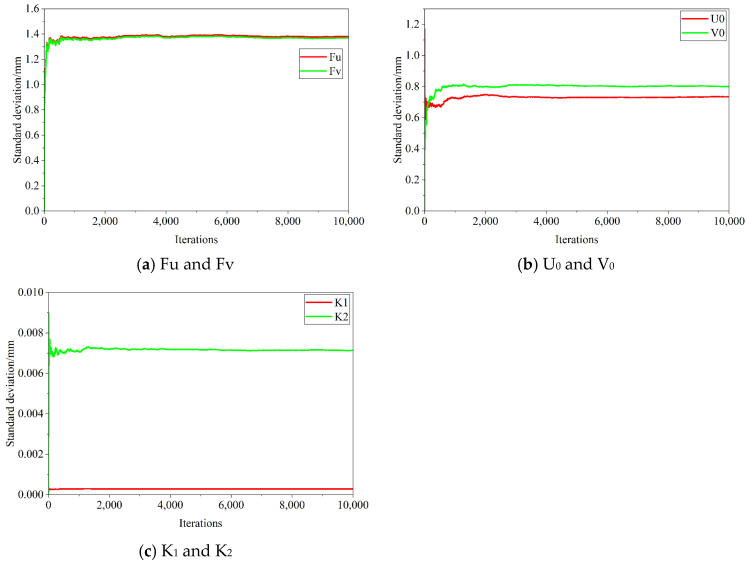
Changes in camera calibration parameters relative to the number of iterations.

According to Jiang’s [23] discussion of the number of iterations of uncertainty Monte Carlo simulation, this paper sets the number of iterations to 10,000. The validity of this attempt will be verified later. First, the uncertainty of corner feature extraction in Table 2 is used as the input uncertainty to calculate the camera calibration uncertainty, as shown in Table 3. Figure 4 shows the changes in camera calibration parameters relative to the number of iterations during 10,000 iterations.

Observing Figure 4, the standard deviation curves of each camera calibration parameter show similar trends; in the first 1000 iterations, the curves change drastically, showing a rapid increase or decrease; in the 1000–2000 iterations, the curves change steadily; and in the 5000–10,000 iterations, the curves tend to stabilize. Taking the change in Fu standard deviation as an example, after 10,000 iterations, the standard deviation value only changes by 0.2‰ relative to the result of 5000 iterations, with almost no drastic change.

**Figure 5:**
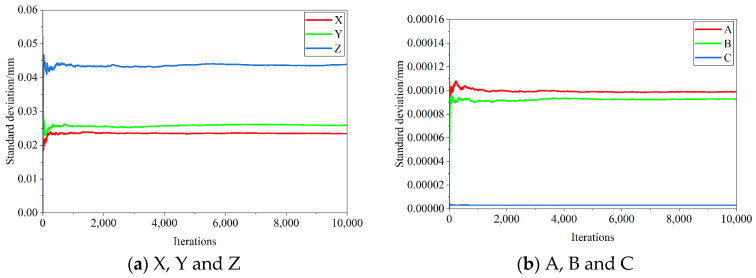
Changes in pose calculation parameters relative to the number of iterations.

Then, the corner feature extraction uncertainty in Table 2 and the camera calibration uncertainty in Table 3 are used as input uncertainties to calculate the pose calculation uncertainty, as shown in Table 4. Due to the small value of radians, 7 decimal places are uniformly retained, the same below. Figure 5 shows the changes in pose calculation parameters relative to the number of iterations during 10,000 iterations.

Observing Figure 5, the curves in Figure 4 and Figure 5 show the same trend. Therefore, based on Figure 4 and Figure 5, it can be concluded that 10,000 iterations are sufficient to stably obtain the uncertainty estimate of this system.

Next, the hand–eye calibration uncertainty is calculated. Because the pose calculation uncertainty in this study is used to calculate the pose of the checkerboard calibration plate in the camera coordinate system, the robot motion uncertainty and the pose calculation uncertainty in Table 4 are directly used as input uncertainties. The Horaud hand–eye calibration method is selected to calculate the hand–eye calibration uncertainty, as shown in Table 5.

Finally, the pose calculation uncertainty in Table 4 and the hand–eye calibration uncertainty in Table 5 are used as input uncertainties to calculate the assembly pose uncertainty, as shown in Table 6.

First, consider the hand–eye calibration uncertainty in Table 5. Although the input uncertainty, robot motion uncertainty, and pose calculation uncertainty are all around 0.03 mm (XYZ), after the hand–eye calibration process, the hand–eye calibration uncertainty in Table 5 reaches 1.5 mm (XYZ). Furthermore, consider the assembly pose uncertainty in Table 6. Although the pose calculation uncertainty is around 0.03 mm (XYZ), the greater hand–eye calibration uncertainty further improves the final assembly pose uncertainty based on the hand–eye calibration uncertainty.

The above uncertainty analysis results reveal that even without considering the robot calibration process, the hand–eye calibration process alone significantly impacts assembly pose uncertainty. By bypassing both the hand–eye and robot calibration processes, with visual measurement at one end and direct connection to the parallel robot positioner’s drive volume at the other, assembly accuracy could be significantly improved. Inspired by this, this study proposes a one-step, end-to-end estimation upgrade for the positioner’s drive volume.

## 4. Positioner Drive Volume One-Step End–End Estimation Upgrade

### 4.1. Positioner Calibration Principle

To facilitate the description of the positioner calibration principle, it is assumed that the joints of the positioner *i* are parallel to the axes of the positioner’s own coordinate system {O_l_}, with trace angles with respect to the global coordinate system {O_g_}. The projections of the three coordinate axes of positioner i under the global coordinate system are

(4) $$e_{x,i}^{g}=\left[\begin{array}{c} \cos\theta_{x,i}\\ \sin\theta_{x,i}\cos\theta_{y,i}\\ \sin\theta_{x,i}\sin\theta_{y,i} \end{array}\right],e_{y,i}^{g}=\left[\begin{array}{c} \sin\varphi_{y,i}\sin\varphi_{z,i}\\ \cos\varphi_{y,i}\\ \sin\varphi_{y,i}\cos\varphi_{z,i} \end{array}\right],e_{z,i}^{g}=\left[\begin{array}{c} \sin\psi_{z,i}\cos\psi_{x,i}\\ \sin\psi_{z,i}\sin\psi_{x,i}\\ \cos\psi_{z,i} \end{array}\right]$$

where the angle between the *x*-axis of positioner *i* and the *x*-axis of the global coordinate system is *θ_x,i_* and the angle between the projection in the *yz*-plane and the *y*-axis is *θ_y,i_*; the angle between the *y*-axis of positioner *i* and the *y*-axis of the global coordinate system is *φ_y,i_*, and the angle between the projection in the *zx*-plane and the *z*-axis is *φ_z,i_*; the angle between the *z*-axis of positioner *i* and the *z*-axis of the global coordinate system is *ψ_z,i_*, and the angle between the projection in the *xy*-plane and the *x*-axis is *ψ_x,i_*.

The coordinate of the origin of positioner *i* under the global coordinate system is $O_{i}^{g}={\left[\begin{array}{ccc} x_{l,i}^{g} & y_{l,i}^{g} & z_{l,i}^{g} \end{array}\right]}^{T}$. The coordinate of the center of the ball hinge *i* under the locator coordinate system is $Q_{i}^{l}={\left[\begin{array}{ccc} x_{q,i}^{l} & y_{q,i}^{l} & z_{q,i}^{l} \end{array}\right]}^{T}$. The coordinates of this point under the global coordinate system are

(5) $$Q_{i}^{g}=O_{i}^{g}+e_{x,i}^{g}x_{q,i}^{l}+e_{y,i}^{g}y_{q,i}^{l}+e_{z,i}^{g}z_{q,i}^{l}$$

The attitude matrix and position vector of the positioner *i* equivalent of the global coordinate system can be expressed as $R_{l,i}^{g}=\left[\begin{array}{ccc} e_{x,i}^{g} & e_{y,i}^{g} & e_{z,i}^{g} \end{array}\right]$, and thus there are

(6) $$Q_{i}^{g}=O_{i}^{g}+R_{l,i}^{g}Q_{i}^{l}$$

The coordinates of the center of the ball hinge in the local coordinate system of the bracket are $Q_{i}^{b}={\left[\begin{array}{ccc} x_{q,i}^{b} & y_{q,i}^{b} & z_{q,i}^{b} \end{array}\right]}^{T}$. The attitude matrix and the position vector of the local coordinate system of the bracket with respect to the global coordinate system are $R_{b}^{g}$, $I_{b}^{g}$, respectively, so that there are

(7) $$Q_{i}^{g}=I_{b}^{g}+R_{b}^{g}Q^{b}$$

where

(8) $$R_{b}^{g}=\left[\begin{array}{ccc} \cos\lambda & -\sin\lambda & 0\\ \sin\lambda & \cos\lambda & 0\\ 0 & 0 & 1 \end{array}\right]\left[\begin{array}{ccc} \cos\beta & 0 & \sin\beta \\ 0 & 1 & 0\\ -\sin\beta & 0 & \cos\beta \end{array}\right]\left[\begin{array}{ccc} 1 & 0 & 0\\ 0 & \cos\alpha & -\sin\alpha \\ 0 & \sin\alpha & \cos\alpha \end{array}\right],I_{b}^{g}=\left[\begin{array}{c} a\\ b\\ c \end{array}\right]$$

The origin of the positioner is generally chosen as the position of the ball hinge when the displacement of each axis of the positioner is 0, i.e., the displacement of each axis of the positioner, $d_{i}$, is equal to the coordinates of the ball hinge in the coordinate system of the positioner, $Q_{i}^{p}$. Setting the displacement of the positioner *i* to be $d_{i}={\left[\begin{array}{ccc} d_{x,i} & d_{y,i} & d_{z,i} \end{array}\right]}^{T}$, there are

(9) $$O_{i}^{g}+R_{l,i}^{g}d_{i}=I_{b}^{g}+R_{b}^{g}Q^{b}$$

We define the mechanism composed of positioner *i* and ball hinge *i* as the pivot chain *i* of the pose adjustment mechanism, and Equation (9) is the kinematics equation of pivot chain *i*. Let $w={\left[\begin{array}{cccccc} \alpha & \beta & \gamma & a & b & c \end{array}\right]}^{T}$, which is the pose attitude of the end (bracket) of the parallel robot; for the pose mechanism composed of n branched chains, the expression of the forward kinematics of the system can be obtained according to Equations (5) and (6):

(10) $$w=h(d_{1},d_{2},\ldots ,d_{n})$$

Due to the manufacturing and installation errors of the positioner and the bracket, there are errors in the kinematic parameters of the system. In order to ensure tuning accuracy, it is necessary to calibrate the kinematic parameters by using external measuring devices, such as laser trackers. Based on the calibrated kinematic parameters, the mutual conversion between the positioner drive volume and the end pose of the parallel robot is realized as the positioner calibration.

### 4.2. Advantages of One-Step End–End Estimation

In the complete hand–eye calibration process, there are a number of factors that can lead to final calibration errors, including visual measurement errors and robot motion errors. In the case of robot calibration, there are also a number of error factors, including the measurement uncertainty of the laser tracker and the angular deviation of the positioner’s own axes. These factors are nonlinear, i.e., the magnitude of these errors does not vary with the input. Most existing calibration methods, especially robot calibration methods, rely on accurate linear models that do not take these errors into account, and therefore lead to inaccurate hand–eye calibration and robot calibration results. Artificial neural networks, on the other hand, due to the use of a series of nonlinear activation functions, can effectively map the nonlinearities and cover the nonlinear errors mentioned above in the network learning phase, resulting in higher accuracy in the final inference phase. This study builds on Zhou’s [16] research by simultaneously covering the parameters of hand–eye calibration and robot calibration in the neural network parameters to improve the final assembly accuracy.

### 4.3. Process of One-Step End–End Estimation

From a purely mathematical perspective, the nine-axis parallel robot system, as a redundant system, theoretically allows for multiple combinations of driving quantities corresponding to the same end-effector pose. However, in practical engineering applications, through a precise positioner calibration process and defined kinematic algorithms, the system is constrained to exhibit a meaningful one-to-one correspondence. This correspondence is established based on three key factors. First, the precise calibration process establishes a unique geometric parameter reference for the system. Second, the inverse kinematics algorithm filters the unique feasible solution from mathematical multiple solutions through defined optimization criteria. Finally, the consistency between physical constraints and the control system ensures the stability of the mapping relationship. Therefore, despite the system’s mathematical redundancy, the constraints imposed in engineering practice result in a deterministic one-to-one correspondence.

The control process of the nine-axis positioner system ensures a precise one-to-one correspondence between end-effector pose and drive commands; when a target end-effector pose is specified, the motion control system calculates a unique inverse kinematics solution using calibrated parameters and generates corresponding nine-axis drive commands. The repeatability and determinism of this process have been verified through extensive experimentation. In practical assembly tasks, the system demonstrates high pose repeatability accuracy, further confirming the uniqueness of the mapping relationship.

This characteristic of parallel robots shares similarities with serial robotic systems: although multiple solutions may exist mathematically for the inverse kinematics of serial robots, practical constraints such as joint limitations and motion planning ensure that each end-effector pose corresponds to a unique combination of joint angles in actual operation. Similarly, parallel robots achieve the transition from mathematical redundancy to engineering uniqueness through calibration and control systems. Therefore, based on the above reasoning, we can conclude that:

(11) $$C=f(A^{*})$$

*C* is the variation of the positioner drive volume, and Equation (12) expresses the one-to-one correspondence between the variation of the positioner drive volume and the variation of the robot end pose. According to Equation (2) and previous research [16], we obtain:

(12) $$A^{*}=f\left(B\right)$$

where A* is the variation of the robot end pose and *B* is the variation of the target object pose in the camera coordinate system. Since the values of the two are transmitted through strict matrix multiplication, they are also one-to-one correspondences.

Mathematically, since both Equations (11) and (12) are bijective, their composite mapping is also bijective, meaning it is one-to-one. In robotic systems, one-to-one correspondence relies not only on mathematical theory but also requires consideration of practical constraints and calibration processes. The visual measurement system establishes a deterministic relationship with the robot end-effector through hand–eye calibration. This calibration process ensures the accuracy and uniqueness of Equation (12). Meanwhile, the mapping from the robot end-effector to the actuator drive of the positioner is guaranteed to be unique through kinematic calibration, as expressed in Equation (11). Consequently, the entire chained process forms a deterministic system after calibration, and the composite mapping naturally maintains one-to-one correspondence. Therefore:

(13) $$\left\{\begin{array}{c} A^{*}\overset{One-to-one}{\to }B\\ C\overset{One-to-one}{\to }A^{*} \end{array}\to \right.C\overset{One-to-one}{\to }B$$

(14) C=f(B)

The functional relationship in Equation (14) is trained and learned using the proposed convolutional neural network to realize the mapping from variation to variation. In the actual assembly process, *B* can be obtained by visual measurement and *C* can be sent to the positioner for docking alignment.

**Figure 6:**
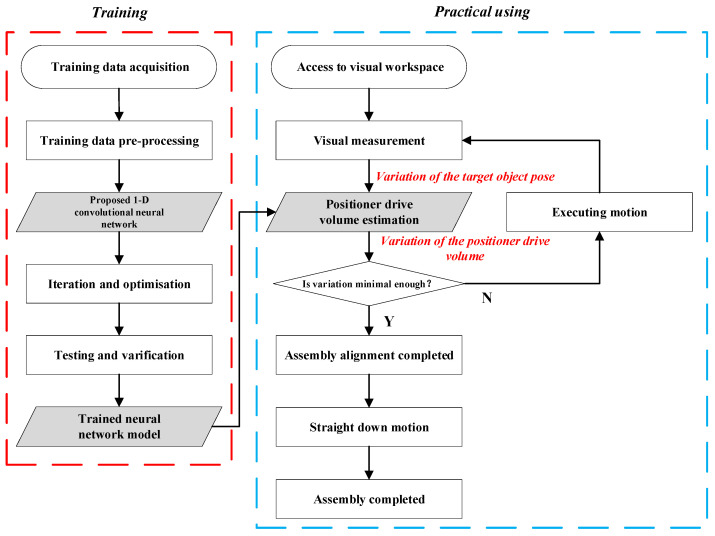
Workflow of one-step end–end estimation.

It is important to emphasize here that we do not obtain any explicit hand–eye relationship matrix and robot calibration parameters throughout the operation, but rather they are implicitly included together in the deep neural network model. Since the parallel robot used in this study consists of three positioners with three axes each, it ends up with a total of nine axes. The entire one-step end–end estimation workflow is shown in Figure 6.

Specifically, the entire process is divided into a training phase and a practical using phase:

Training phase:

1. Acquire and pre-process data.
2. Design suitable convolutional neural network (often requires a lot of experimentation).
3. Iterative optimization to complete the training.
4. Test and validate to obtain the trained network model.

Practical using phase:

1. Access to visual work range.
2. Visual measurement to obtain the variation of the target object pose in the camera coordinate system.
3. Based on the trained network model, input the variation and output the variation of the positioner drive volume.
4. Determine whether the variation is small enough, if yes, then finish adjusting the pose.
5. If not, adjust the pose and repeat step 2.

It should be noted that after obtaining the variation of the positioner drive volume, in order to prevent motion interference, this study uses Chu’s [33] method for tuning.

## 5. Proposed One-Step End–End Estimation Convolutional Neural Network (OECNN)

### 5.1. Training Data Acquisition and Processing

The mechanical parameters of the parallel robot used in this study are presented in Table 7.

**Figure 7:**
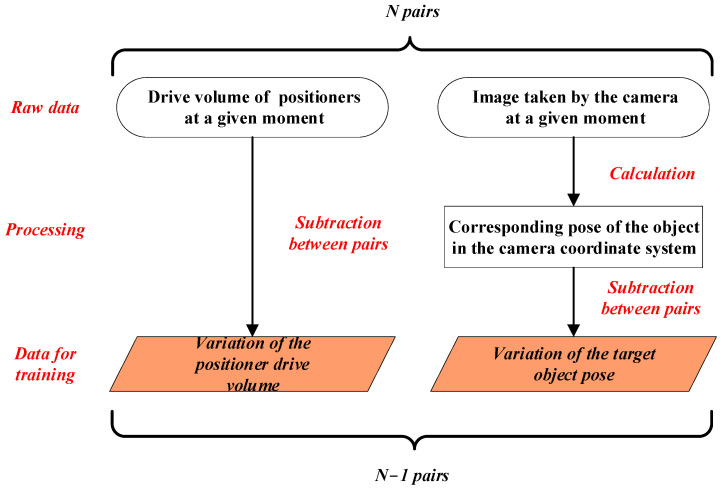
Training data pre-processing process.

We use the Latin Hypercube Sampling (LHS) method [47] for sampling planning to ensure that the sampling points can reflect the state of the entire robot workspace as much as possible [48]. A rectangular parallel-hexagonal region with dimensions of 50 mm × 30 mm × 30 mm and attitude angles of 10°, 4°, and 3°, respectively, is selected as the sampling space. According to the LHS method, 400 position coordinates and 250 attitude angles are randomly generated in the sampling space, and then they are arranged and combined to obtain the attitude of 100,000 sampling points. We send the sampling point poses obtained by the above sampling method to the control system of the parallel robots, control the robots to move to the commanded poses in sequence, directly record the drive volume readings of the three positioners with a total of nine axes at this moment, and then collect the corresponding 100,000 photos of the calibration plate with an industrial camera. The training data preprocessing flow is shown in Figure 7. It is important to emphasize that this study uses a calibration plate in the training data production stage. This is because the camera’s measurement accuracy of the calibration plate’s pose, given its known dimensions, is high, and also because camera measurement of the calibration plate is highly efficient. In actual use, the monocular camera will capture and measure the pose of the actual assembly features, not the calibration plate. Furthermore, using deep learning super-resolution and pose optimization algorithms, the camera’s accuracy in measuring the actual assembly features will closely approximate the accuracy of the camera’s measurement of the calibration plate. The data used for training and inference is not images of the calibration plate or actual assembly features, but rather the target pose calculated from those images. Therefore, using a calibration plate at this stage will not result in a failure to cover the actual scene.

The specific process of training data pre-processing is as follows:

1. Pre-calibrate the camera to get the internal parameters of the camera.
2. Combine the camera internal parameters with 100,000 calibration plate photos to calculate the pose of 100,000 corresponding calibration plates in the industrial camera coordinate system.
3. Pair the above 100,000 poses with the 100,000 corresponding positioner drive volume to get 100,000 pairs of data.
4. Divide the training data set and test data set by a certain ratio. In this study, a ratio of 9:1 was chosen, i.e., 90,000 pairs of pose data were used for training and 10,000 pairs of pose data were used for testing.
5. The order of the 90,000 pairs of training data is disordered, and the difference between the front and back is made to form the pose variation data for deep learning network training.

### 5.2. Basic Architecture of OECNN

**Figure 8:**
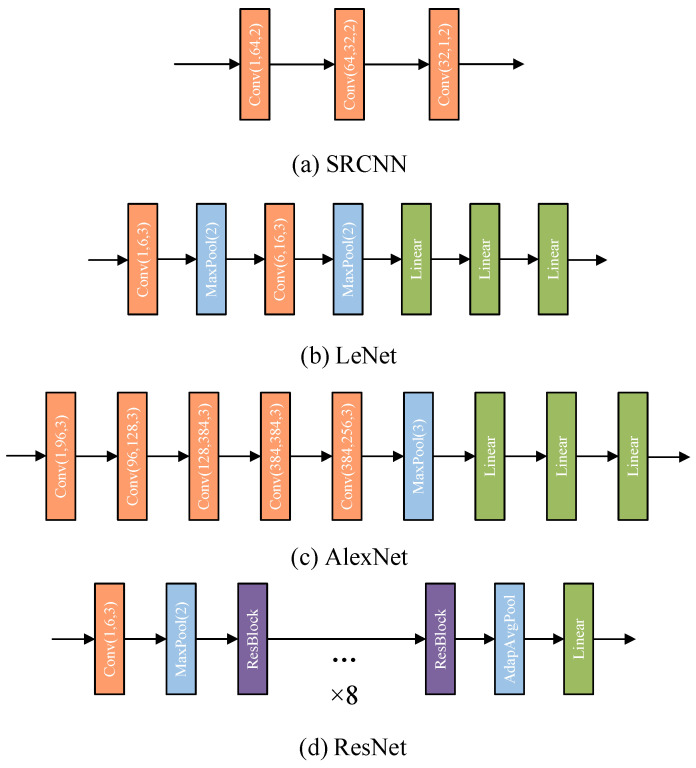
Four new network architectures for drive volume estimation.

The one-step end–end estimation convolutional neural network (OECNN) pro-posed in this study is inspired by four classical network architectures, SRCNN [49], LeNet [50], AlexNet [51] and ResNet [52]. In order to apply these four classical net-work architectures to be able to apply to the driver volume estimation task in this paper, which satisfies the requirement of 6 dimensions for inputs and 9 dimensions for outputs, their network structures and hyperparameters are fine-tuned as shown in Figure 8.

**Figure 9:**
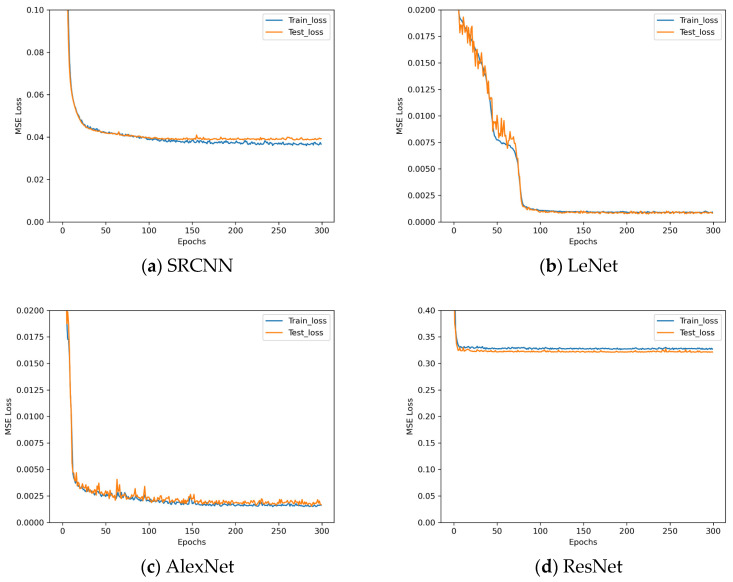
Loss decent curves of four new network architectures.

In Figure 8, Conv(I, o, k) denotes the convolutional layer, I denotes the number of channels in the input of the convolutional layer, o denotes the number of channels in the output of the convolutional layer, and k denotes the kernel size. Linear denotes the linear layer. MaxPool(k) denotes the maximum pooling layer. AdapAvgPool denotes the adaptive averaging pooling layer. The four networks are trained on the acquired data using a improved MSE loss as the loss function, the epoch was set to 300 and the loss descent curves during training are shown in Figure 9.

Since the task of this study is high-precision assembly, according to the definition of MSE loss, it is essentially an expression of assembly posing accuracy, meaning that MSE loss has a decisive impact on assembly accuracy. Therefore, this paper establishes the standard of evaluating network quality based on MSE loss. Analyzing the results in Figure 9, we can observe that the LeNet* architecture has the minimum training loss, while ResNet* has the maximum training loss. Although AlexNet* has results that are very closed to LeNet*, it suffers from a light overfitting phenomenon during training. So, the LeNet* architecture is chosen as the basis for the proposed OECNN architecture in this paper. Owing to the slight overfitting observed in AlexNet*, to ensure thoroughness, experiments are first conducted on LeNet* using both dropout and L2 regularization methods to validate the impact of overfitting mitigation strategies on its training. The dropout rates employed in these experiments are detailed in Table 8.

**Figure 10:**
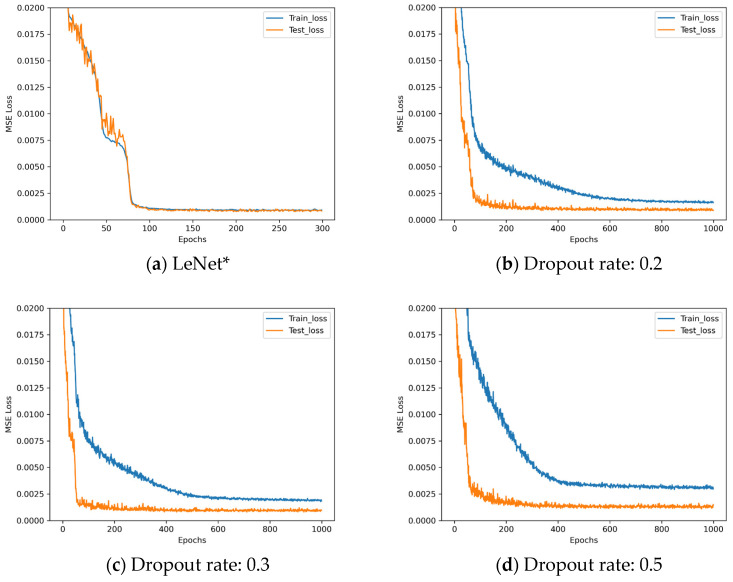
Loss descent curves in dropout rate experiments.

The loss descent curves of these four architectures with different dropout rates during the training process are shown in Figure 10.

Observing Figure 10, LeNet* demonstrates poor performance when employing the dropout method. Analysis indicates that LeNet*, being a relatively shallow convolutional neural network with inherently limited parameters, may suffer from underfitting when dropout is applied. The L2 regularization parameters employed in these experiments are detailed in Table 9.

**Figure 11:**
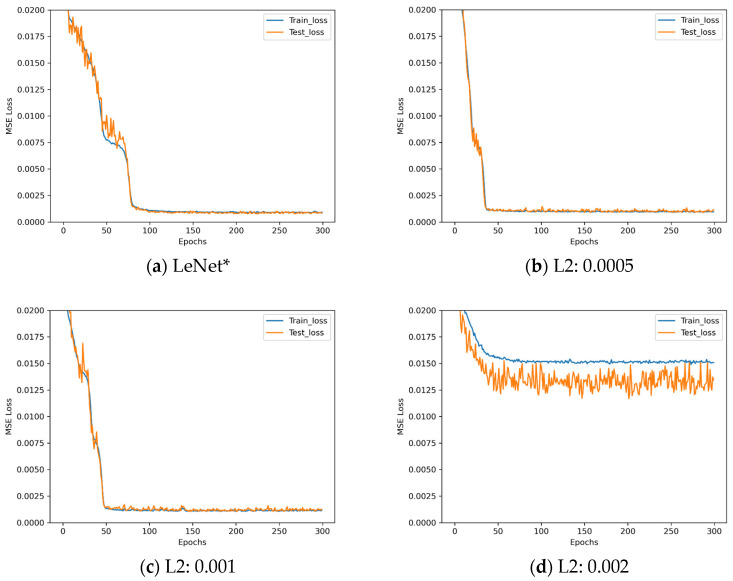
Loss descent curves in L2 regularization parameter experiments.

The loss descent curves of these four architectures with different L2 regularization parameters during the training process are shown in Figure 11.

Observing Figure 11, when the regularization parameter is set to 0.0005, the network training performance resembles that of the original network. Although convergence occurs earlier, the fluctuations in the curve become more pronounced. Conversely, when the regularization parameter is set to a larger value, the network training performance deteriorates. Analysis indicates that L2 regularization constrains model complexity by penalizing large weights. However, for the inherently uncomplicated LeNet*, this constraint may impose excessive restrictions, leading to underfitting and consequently poor performance. Based on the aforementioned experiments, LeNet* requires no additional methods to counteract overfitting.

### 5.3. Determination of Optimal Network Architecture

The following training experiments are used to analyze and discuss the optimal architecture of the OECNN network. Firstly, the number of convolutional layers of the network is increased, and the experiment parameters set for the network layer control experiments are shown in Table 10.

**Figure 12:**
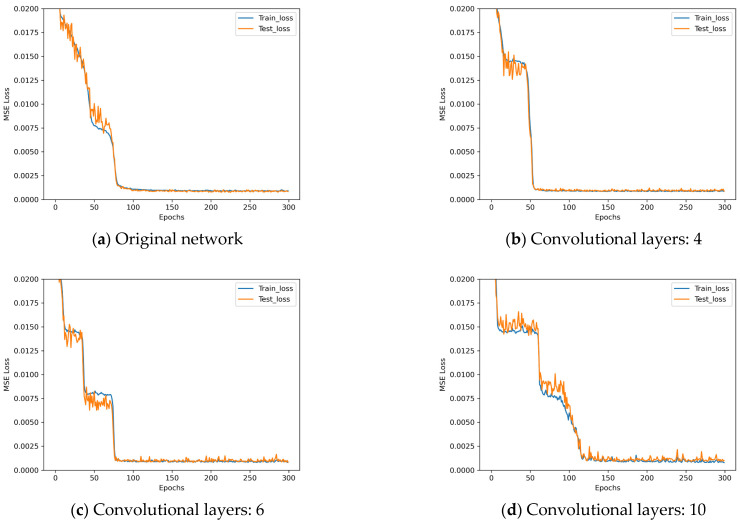
Loss descent curves in network layer control experiments.

The loss descent curves of these four architectures with different number of network layers during the training process are shown in Figure 12.

Analyzing the results in Figure 12, it can be observed that there is no significant improvement in the performance of the network when the number of convolutional layers in the network is increased from 2 to 4. However, when the number of convolutional layers in the network is further increased to 6, a slight overfitting phenomenon can be observed. Finally, when the number of convolutional layers is increased directly to 10, a significant overfitting phenomenon occurs. So, the number of convolutional layers in the network is determined to be 2 for the proposed OECNN architecture.

Next, the analysis of the need for pooling layers in the original network architecture is continued with control experiments. The experiment parameters for the pooling layer control experiments are set up as shown in Table 11.

**Figure 13:**
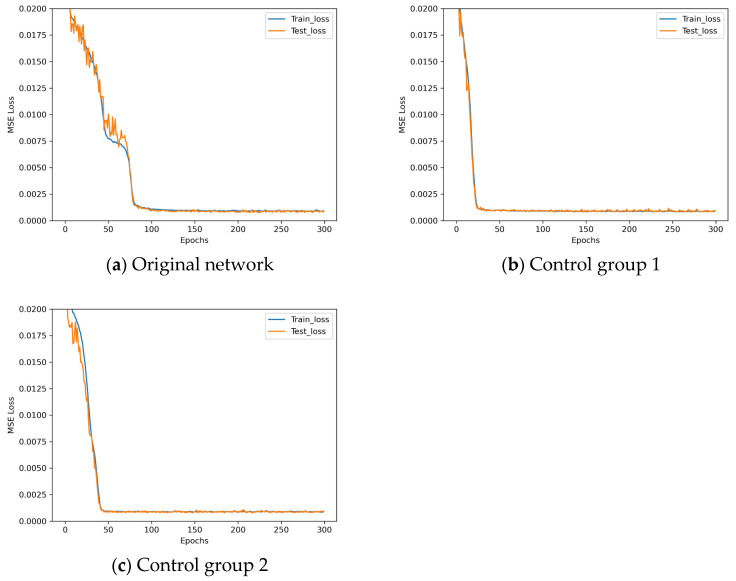
Loss descent curves in pooling layer control experiments.

The loss descent curves of these three architectures with different numbers of pooling layers during the training process are shown in Figure 13.

Analyzing the results in Figure 13, it can be observed that removing the pooling layer at the front end of the network structure and retaining the pooling layer in front of the linear layer does not cause a significant change in the loss of final convergence of the network, but it converges significantly better and earlier. In contrast, when all pooling layers in the network structure are removed, the network converges more slowly. Below is a detailed analysis. In traditional image processing, pooling layers primarily serve functions such as spatial downsampling, translation invariance, and feature compression. However, when processing 6-D pose vectors, these functions require reconsideration. Within the 6-D pose vector, each dimension carries specific physical meaning, and strict geometric constraints exist between dimensions. Premature pooling operations can disrupt this fine-grained structural information. Removing pooling layers from the front end of the network preserves the original physical meaning of the 6-D vector, enabling convolutional layers to directly learn local correlations between dimensions. Retaining pooling layers at the end extracts the most discriminative global patterns, providing compact feature representations for regression tasks. In other words, the aforementioned experimental phenomena reveal the unique mechanism of pooling layers in processing low-dimensional sequential data. Removing the front-end pooling layer preserves the integrity of original features and promotes more efficient gradient flow, thereby accelerating convergence. Retaining the terminal pooling layer provides essential feature aggregation and regularization, ensuring training stability.

**Figure 14:**
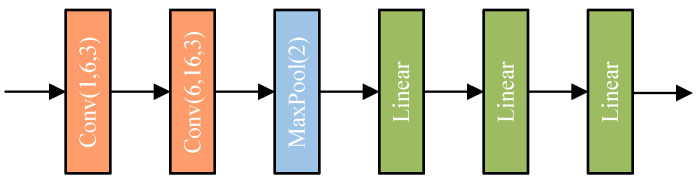
Optimal architecture of OECNN.

So, the architecture of OECNN is determined to be the one with the pooling layer at the end of the network and without the pooling layer at the front end, as shown in Figure 14.

Subsequently, experiments are conducted on the convolution kernel size, activation function, and normalization method employed in OECNN. The experiment parameters for the convolution kernel size control experiments are set up as shown in Table 12.

**Figure 15:**
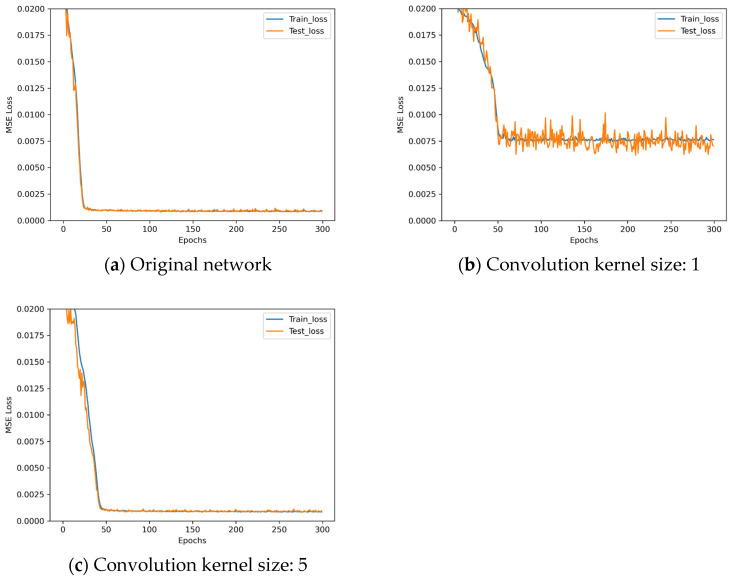
Loss descent curves in convolution kernel size control experiments.

The loss descent curves of these three architectures with different convolution kernel sizes during the training process are shown in Figure 15.

Observe Figure 15: when the convolution kernel size is 1, the network performs poorly. When the kernel size is 5, although the training results are similar to those with a kernel size of 3, convergence is slower. Therefore, the convolution kernel size remains unchanged. The experiment parameters for the activation function control experiments are set up as shown in Table 13. Three additional activation functions are selected for comparison based on data type.

**Figure 16:**
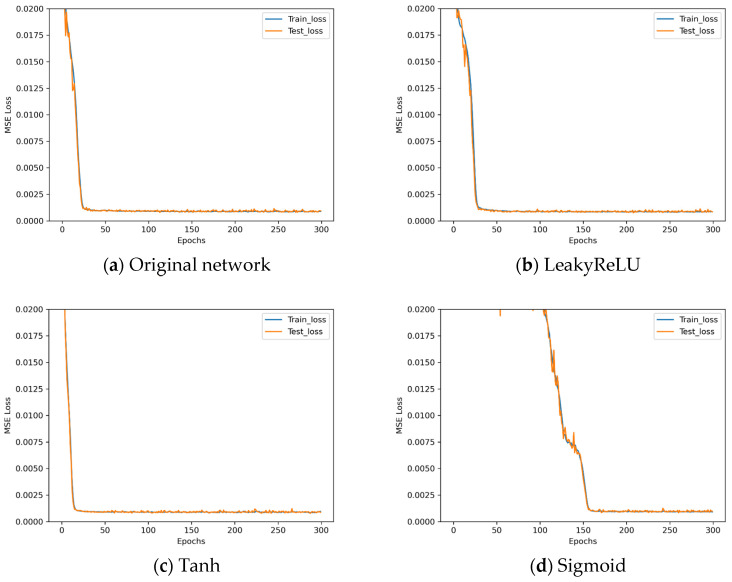
Loss descent curves in activation function control experiments.

The loss descent curves of these four architectures with different activation functions during the training process are shown in Figure 16.

Observing Figure 16, the performance when using LeakyReLU is similar to that of ReLU, whilst the network converges more rapidly when employing Tanh. Beyond this, however, no significant differences in performance are discernible. The results obtained using Sigmoid prove less favourable. Given that Tanh’s computational complexity is several times that of ReLU without yielding any discernible improvement in performance, the activation function remains unchanged at the classical ReLU. The experiment parameters for the normalization technique control experiments are set up as shown in Table 14.

**Figure 17:**
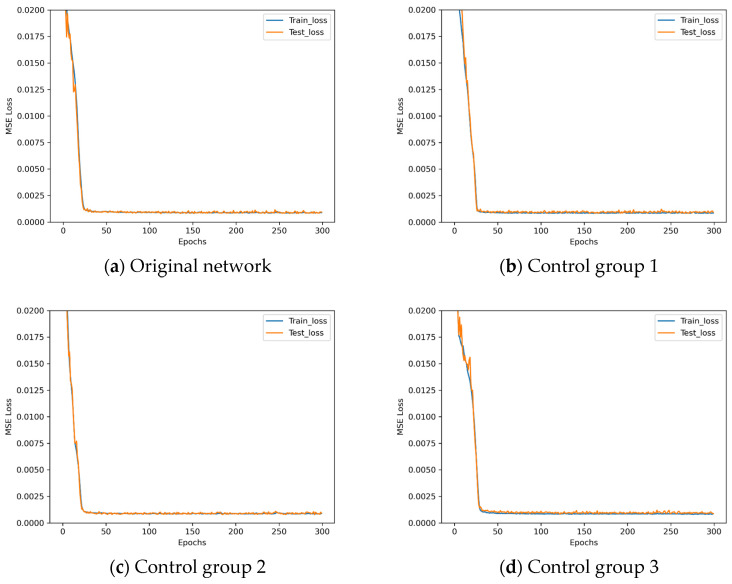
Loss descent curves in normalization technique control experiments.

The loss descent curves of these four architectures with different normalization techniques during the training process are shown in Figure 17.

Observing Figure 17, normalization techniques do not yield improvements in training outcomes and may even result in negative optimization. Analysis indicates that for shallow networks such as OECNN, the gradual nature of data distribution changes means the benefits introduced by normalization layers are insufficient to offset the increased computational complexity they introduce. Therefore, OECNN does not employ normalization techniques.

### 5.4. Determination of Optimal Network Hyperparameters

Next, we conduct further experiments to analyze and discuss the optimal hyperparameters of OECNN. Firstly, we conduct control experiments of the convolutional layer parameters. Since the input data is in the form of 1 × 6, we did not modify the convolutional kernel size of the convolutional layers, and the experiment parameters set is shown in Table 15.

**Figure 18:**
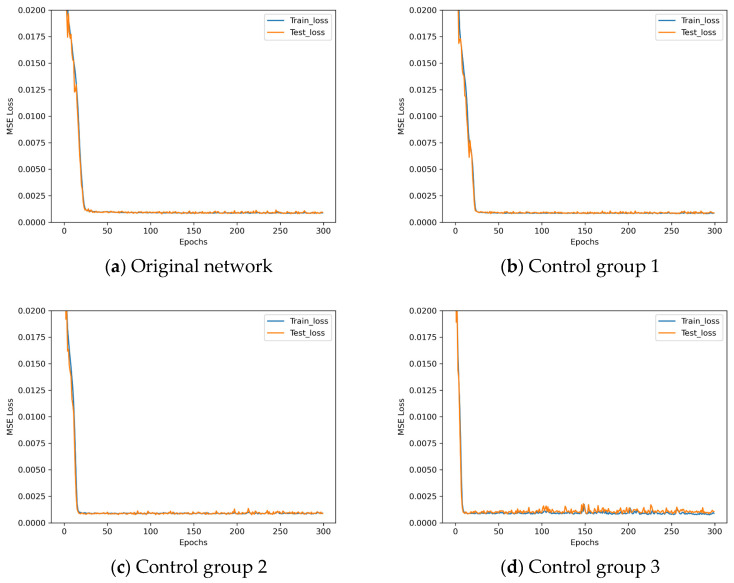
Loss descent curves in convolutional layer parameter control experiments.

The loss descent curves of these four architectures with different convolutional layer parameters during the training process are shown in Figure 18.

Analyzing the results presented in Figure 18, it can be observed that increasing the number of input channels and output channels in the convolutional layers cannot significantly improve training results, instead, there is a risk of worsening the results. Therefore, the decision is made not to increase the number of channels in the convolutional layers.

The following are learning rate control experiments with the experiment parameters set as shown in Table 16.

**Figure 19:**
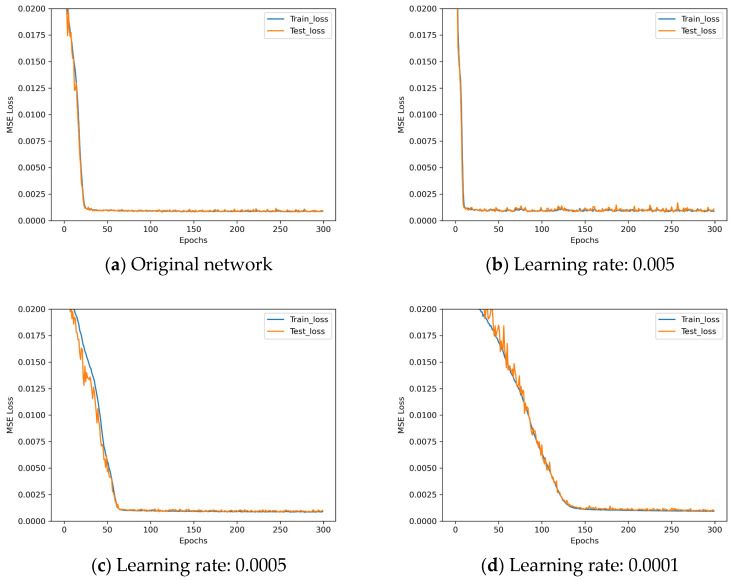
Loss descent curves in learning rate control experiments.

The loss descent curves of these four architectures with different learning rates during the training process are shown in Figure 19.

After analyzing the results in Figure 19, it can be observed that when the learning rate is increased to 0.005, the training results begin to deteriorate, while reducing the learning rate to 0.0005 and 0.0001 shows slower training convergence. Based on these results, a learning rate of 0.001 is chosen as it shows the best performance.

Finally, epoch control experiments are performed, and the experiment parameters set are shown in Table 17.

**Figure 20:**
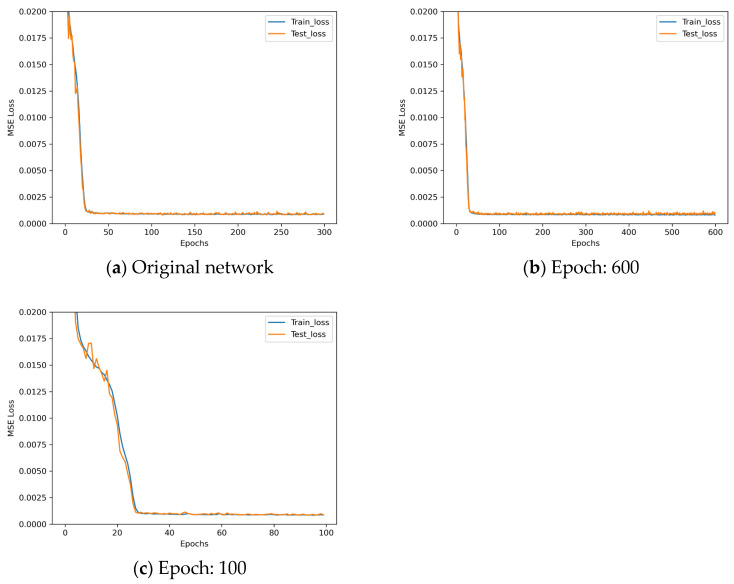
Loss descent curves in epoch control experiments.

The loss descent curves of these three architectures with different epochs during the training process are shown in Figure 20.

After analyzing the results shown in Figure 20, it can be observed that increasing the number of epochs to 600 leads to slight deterioration in training results, while reducing the number of epochs to 100 results in a better fitted loss. Therefore, in this paper, the final epoch is set to 100.

After conducting the experiments discussed earlier, the optimal hyperparameters for the OECNN network have been determined and are presented in Table 18. Finally, with the hyperparameters set as in Table 18, the OECNN network is trained to obtain the final trained model.

## 6. Experiment

### 6.1. Positioner Drive Volume Estimation Accuracy Experiment

To investigate the generalization ability of OECNN, we acquired 10,000 new data sets for positioner drive volume estimation accuracy experiments using the method described before. In these experiments, we compare the performance of the trained OECNN model with Tsai’s hand–eye calibration method [35], laser tracker-based direct measurement method [53], and Zhou’s PECNN network [16].

Using Tsai’s hand–eye calibration method and laser tracker direct measurement method to first obtain the hand–eye relationship matrix, then obtain the corresponding robot end pose based on the inputs in the dataset, and finally use the pre-obtained robot calibration parameters to obtain the variation of the positioner drive volume; using the trained PECNN network, the corresponding robot end pose is obtained directly based on the inputs in the dataset, and continue to use the pre-obtained robot calibration parameters to get the variation of the positioner drive volume. In contrast, the OECNN proposed in this paper can directly obtain the variation of the positioner drive volume to compare with the dataset labels.

All 10,000 sets of new data generated were used to verify the accuracy of the above positioner drive volume estimation, and Equation (15) was used as an error expression to determine the accuracy.

(15) $$Error=\sqrt{\sum_{i=1}^{3}{\left(X_{i}-x_{i}\right)}^{2}+{\left(Y_{i}-y_{i}\right)}^{2}+{\left(Z_{i}-z_{i}\right)}^{2}}$$

**Figure 21:**
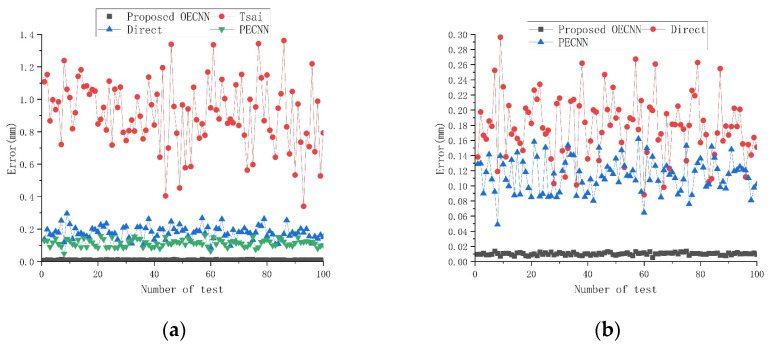
Results of positioner drive volume estimation accuracy experiment: (**a**) Four methods including proposed OECNN, Tsai, direct and PECNN; (**b**) Three methods including proposed OECNN, direct and PECNN.

*X_i_*, *Y_i_*, *Z_i_* represent the true value, i.e., the label in the test dataset, and *x_i_*, *y_i_*, *z_i_* represent the calculated value. The error results are shown in Figure 21 (for ease of display, the horizontal axis is compressed from 10,000 to 100) and Table 19.

After analyzing the above results, it can be concluded that OECNN achieves an accuracy of close to 0.01 mm for the estimation of the positioner drive volume, which is significantly better than the other methods. Although PECNN achieves a high accuracy of pose estimation, it is superior to the errors present in the robot calibration, resulting in a final accuracy of more than 0.1 mm. The traditional hand–eye calibration method is superior to the more error links involved, so it has the lowest accuracy.

**Figure 22:**
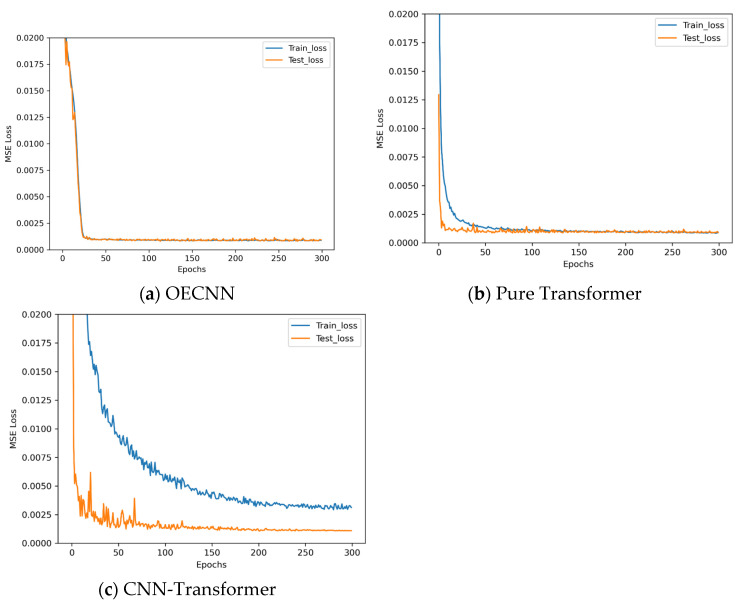
Loss descent curves in Transformer experiments.

To further compare OECNN with state-of-the-art attention-based network architectures represented by Transformers, we designed both a pure Transformer encoder model architecture tailored for this task and a CNN-Transformer hybrid model architecture. The pure Transformer encoder model adopts a sequence-to-sequence regression architecture based on self-attention, specifically designed for the pose mapping task from 6-D input to 9-D output. It employs an encoder-only structure comprising four core components: an input projection layer, a positional encoding module, a multi-layer Transformer encoder stack, and an output regression layer. The CNN-Transformer hybrid model is more innovative, aiming to combine the local feature extraction capability of convolutional neural networks with the global dependency modeling advantage of Transformers. It adopts a layered processing strategy, with the overall architecture comprising four core modules: CNN feature extractor, positional encoding module, Transformer encoder stack, and output regression layer. Both models were trained using the training data, with the training results shown in Figure 22.

Observing Figure 22, the training results are disappointing. The pure Transformer architecture shows no advantage over OECNN, and the loss curve exhibits slightly increased fluctuations. Meanwhile, the CNN-Transformer architecture, though theoretically combining the strengths of both, yields poor training outcomes. Analysis of the experimental phenomena reveals that the sequence modeling advantages of Transformer-like architectures cannot be leveraged in 6-D input tasks, instead introducing unnecessary complexity and optimization challenges. The spatial locality and translation invariance priors inherent in CNNs align exceptionally well with the pose estimation task, forming the core reason for OECNN’s superior performance. Simply concatenating CNN and Transformer components may cause feature representation conflicts and optimization imbalances, necessitating carefully designed fusion mechanisms.

**Figure 23:**
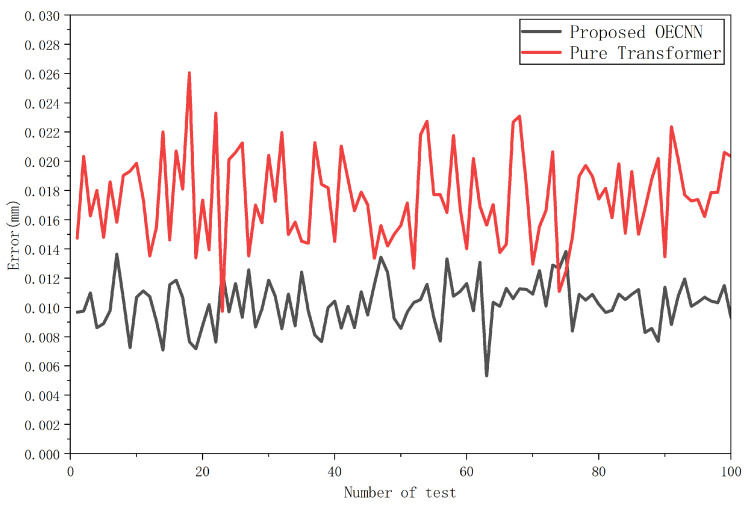
Accuracy results of OECNN and pure Transformer.

Based on the above experiments, we conclude that for the current scale estimation task, the fully optimized OECNN architecture based on CNN provides the optimal balance of performance, efficiency, and stability. This also offers crucial guidance for selecting architectures for similar low-dimensional precision regression tasks: more advanced architectures are not necessarily better suited for specific tasks. Architecture design must be closely aligned with the problem characteristics and practical constraints. The following accuracy experiments are conducted using the trained Pure Transformer and OECNN, with results shown in Figure 23.

Observing Figure 23, the pure Transformer shows no advantage over OECNN, and its network complexity is significantly higher than that of OECNN. Based on the above findings, this demonstrates the superiority of OECNN for handling this task.

### 6.2. Practical Application

**Figure 24:**
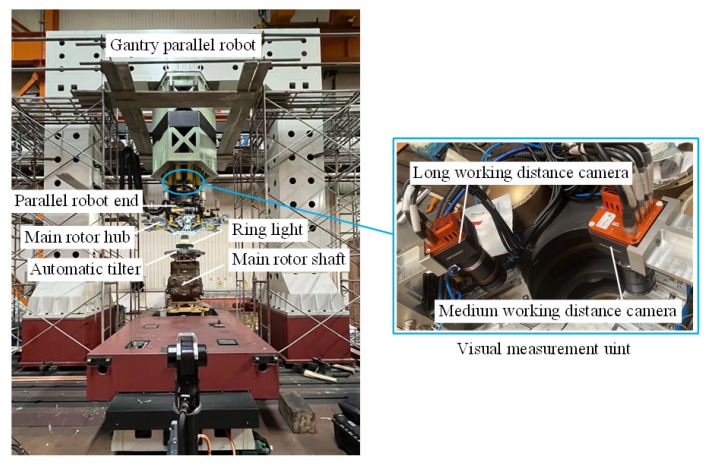
Actual assembly site of the lift system.

Through relevant research, the research results of this paper have been successfully applied to the automated assembly of the lift systems of a medium-sized helicopter and a large helicopter. The actual assembly site is shown in Figure 24.

As shown in Figure 24, at the actual lift system assembly site, due to the large size of the lift system, a gantry-type parallel robot is used as the actuator, with its end-point capable of six degrees of freedom. During the assembly process, the main rotor shaft is fixed in position. The end-point of the parallel robot grips the automatic tilter and main rotor hub, respectively. A visual measurement unit mounted on a moving platform at the end-point measures the pose and guides the installation. The visual measurement unit includes a long- and medium-range cameras, used to measure the pose of the main rotor shaft at a distance of 1000 mm and the tilter/main rotor hub at a distance of 2000 mm, respectively. The cameras are mounted on two high-precision translation stages. When image acquisition is required, the translation stage pushes the camera to the center of the moving platform and then returns it after acquisition is complete.

**Figure 25:**
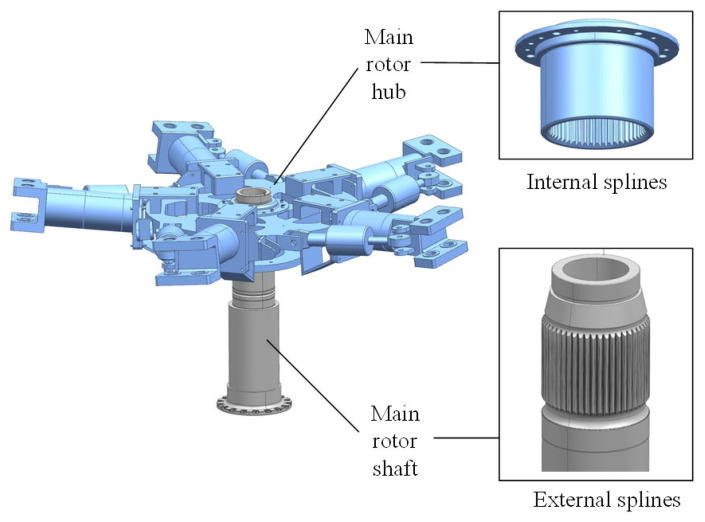
The way the main rotor shaft and the main rotor hub fit together.

In large and medium-sized helicopters, the automatic tilter is connected to the precision matching surface of the main rotor shaft through a ball joint. The main rotor hub and the main rotor shaft are connected by a spline, and the fitting clearance of the spline is less than or equal to 0.05 mm. This places very high demands on assembly accuracy, as shown in Figure 25.

The uncertainty analysis method proposed in this paper played a significant role in the ultimate successful assembly of this project. First, after completing the hardware platform shown in Figure 25, to preliminarily evaluate the accuracy of the entire assembly system, we used this method to obtain the relevant input uncertainties and conducted a preliminary analysis of the system uncertainty. The uncertainty analysis results are shown in Table 15.

As shown in Table 20, the initial accuracy of the assembly system is far from the actual assembly accuracy requirement. Subsequently, the proposed OECNN was applied to this actual assembly, and the proposed method was used to re-obtain the relevant input uncertainties. The final assembly uncertainty was further analyzed, and the results are shown in Table 21.

**Figure 26:**
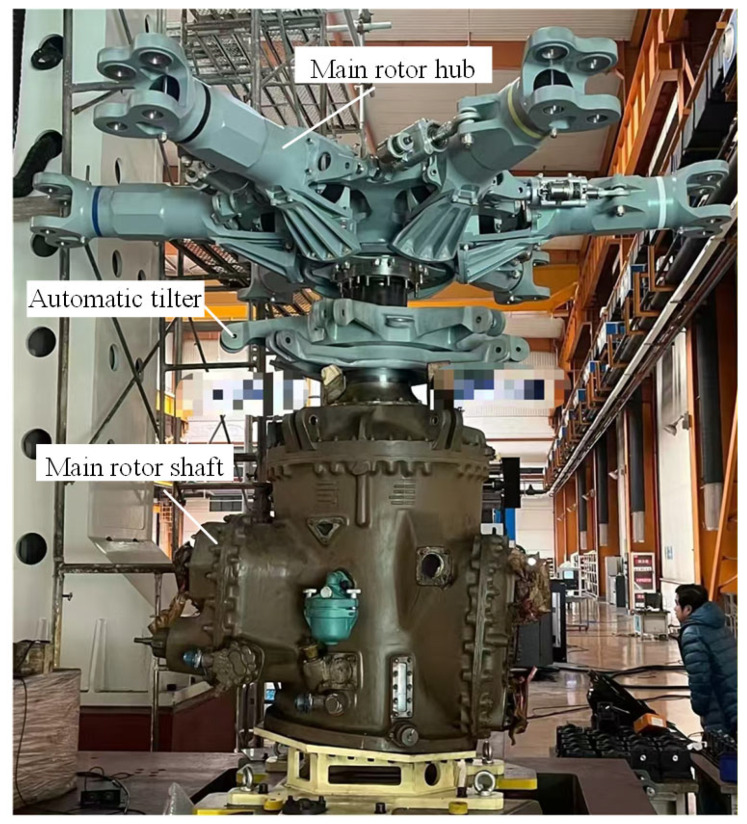
Helicopter lift system after completion of assembly.

The data in Table 21 show that the current assembly system accuracy has basically met the assembly accuracy requirements, that is, the assembly system in Figure 24 is already able to be assembled. Under the pre-assembly analysis based on the data in Table 21, the helicopter lift system was successfully assembled, as shown in Figure 26.

Through the above practical applications, the feasibility of the proposed method for uncertainty analysis of complex systems is first demonstrated, which is especially suitable for large-scale assembly systems with high precision requirements. More importantly, the accuracy of the OECNN proposed in this paper is verified, and it is proved that it can be successfully applied to the assembly of large-scale aviation hole-shaft structures.

### 6.3. Method Applicability Experiment

**Figure 27:**
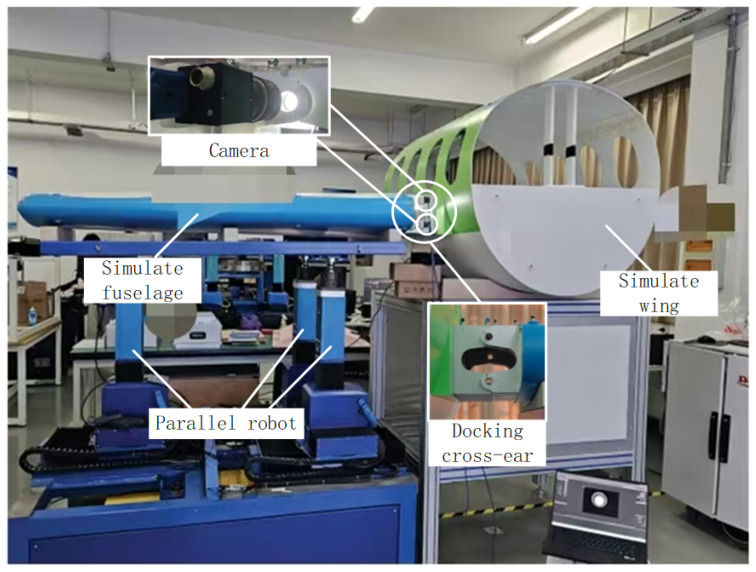
Aircraft wing and fuselage assembly.

**Figure 28:**
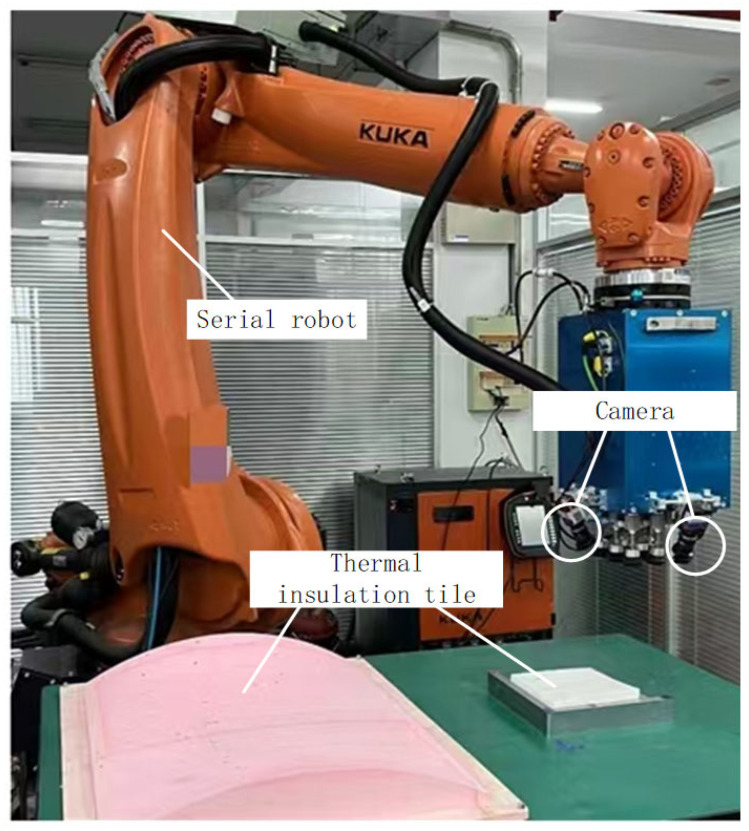
Space shuttle thermal insulation tile assembly.

To validate the applicability of the proposed method across different systems of parallel robots and serial robots, the complete methodology is successfully applied to aircraft wing-fuselage assembly, as illustrated in Figure 27, and to space shuttle thermal insulation tile assembly, as depicted in Figure 28.

**Figure 29:**
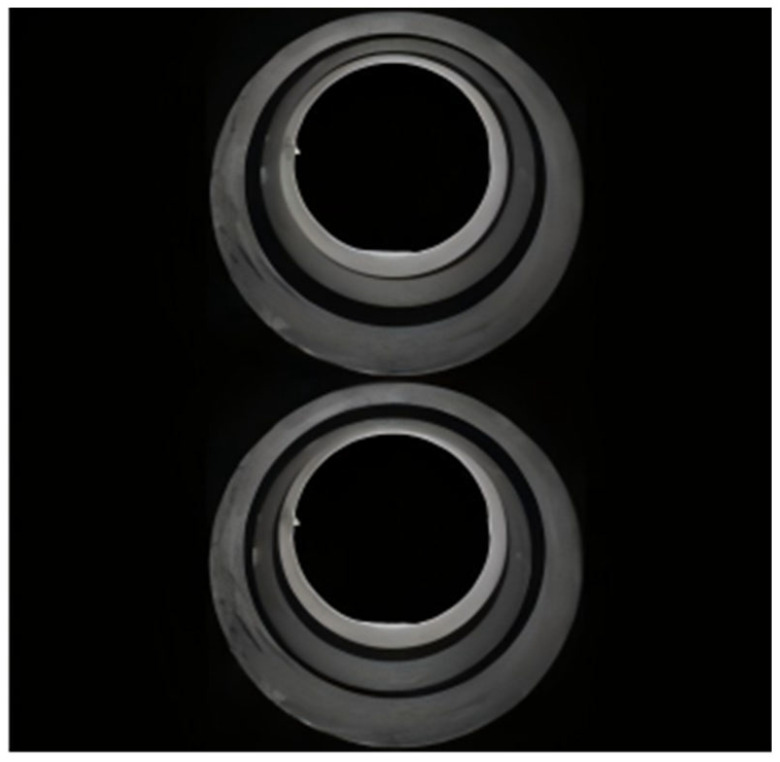
Crossed-ears image.

As illustrated in Figure 27, the aircraft wing and fuselage are assembled via cross-ears. During assembly, the fuselage remains stationary while a parallel robot manipulator lifts and moves it. Two industrial cameras measure the ears’ pose, with captured images shown in Figure 29. In this configuration, constrained camera mounting positions complicate hand–eye calibration. Consequently, the proposed method establishes a mapping relationship between the pose variations measured by the two cameras and the drive variations across the nine axes of the parallel robot’s three positioners. It should be noted that, owing to the presence of two cameras, the OECNN input comprises a twelve-dimensional vector, while its output is a nine-dimensional vector. Employing the method described herein, the successful assembly of the aircraft wing and fuselage is achieved.

As illustrated in Figure 28, a typical KUKA six axes serial robot is employed to execute the thermal insulation tile assembly task for the space shuttle. A vacuum suction cup is fitted at the robot’s end-effector for gripping the tiles, while four cameras are mounted concurrently. Utilizing the methodology presented herein, a mapping relationship is established between the pose variations measured by the four cameras and the drive variations across the serial robot’s six axes. With four cameras employed, the OECNN receives a twenty-four-dimensional input vector and produces a six-dimensional output vector. This approach successfully completed the thermal tile assembly task. In summary, the method applicability experiment confirms that it is suitable not only for typical configurations but also for other multi-camera guided assembly tasks utilizing serial/parallel robots.

## 7. Conclusions

In this study, we first proposed an uncertainty analysis method for a monocular vision-guided assembly system. We introduced a monocular visual measurement system for assembly poses, identified an uncertainty transfer path, and implemented an uncertainty analysis scheme. Through repeated experiments, we obtained input uncertainty values, analyzed the uncertainty of the assembly system, and obtained and analyzed relevant conclusions. Inspired by these conclusions, we further proposed a one-step, end-to-end estimation method based on deep learning. This method incorporates both robot and hand–eye calibration processes, addressing the problem of low final assembly accuracy despite high visual measurement accuracy due to the influence of robot and hand–eye calibration. We first collected and preprocessed a dataset for network training. We then selected four classic network architectures for experimentation and chose the most effective one as the base network for this task. We then conducted a series of experiments to determine the optimal architecture and hyperparameters for the proposed network. Accuracy tests of the localizer-driven quantity estimation and practical applications demonstrated that the proposed method has high accuracy and effectiveness, and can be applied to real-world assembly applications.

While the OECNN network proposed in this paper has proven effective, there is still potential for further improvement. Future research will focus on three core directions for in-depth exploration:

First, at the perception level, integrating 3D vision cameras (including stereo vision, time-of-flight, or structured light sensors) will involve developing novel 3D-OECNN hybrid architectures. These will combine the learning-based accuracy advantages of current monocular vision with the spatial perception capabilities of 3D point clouds. Through multimodal sensor fusion technology, the system’s adaptability to environmental variations (such as lighting changes and vibration interference) will be enhanced, improving absolute positioning accuracy in large-scale operations and optimizing computational efficiency in dynamic real-time control scenarios. Second, future work will systematically incorporate modeling of long-term influencing factors, including camera intrinsic parameter shifts due to thermal drift, accuracy degradation caused by actuator mechanical wear, and performance degradation analysis across multiple assembly cycles. By establishing environment-performance mapping models and accelerated aging test platforms, online compensation for parameter drift and predictive maintenance will be achieved, ensuring system reliability during prolonged operation. Finally, addressing bottlenecks in safety-critical applications, we will deepen research into the explainability of attitude error causes. Using a hierarchical error traceability framework, total error will be decomposed into components such as visual measurement, hand–eye calibration, and robotic motion. Developing out-of-distribution detection algorithms and fault-tolerant control mechanisms to enhance the system’s anomaly posture recognition and fault mitigation strategies. This will deliver intelligent assembly solutions featuring high precision, transparency, and robustness for high-risk sectors like aerospace. The synergistic advancement of these directions will propel vision-guided assembly systems from laboratory validation to industrial deployment, ultimately enabling reliable implementation of smart manufacturing in safety-critical scenarios.

## Figures and Tables

**Table 1:** Camera parameters in repeated tests.

Parameter Category	Specific Parameters
Camera model	MV-CH650-90XM-F-NF (Hikrobot, Hangzhou, China)
Lens model	Hikrobot MVL-LF8040M-F, f = 80 mm (Hikrobot, Hangzhou, China)
Data Interface	CoaXPress
Resolution	9216 × 7000
Sensor	GMAX3265 (Gpixel, Changchun, China)
Pixel size	3.2 µm × 3.2 µm
Frame rate	31.5 fps

**Table 2:** Corner feature extraction uncertainty.

Feature Extraction Parameters	Uncertainty
u (pixel)	0.0508
v (pixel)	0.0429

**Table 3:** Camera calibration uncertainty.

Camera Calibration Parameters	Uncertainty
Fu (mm)	1.3617
Fv (mm)	1.3503
U_0_ (pixel)	0.7325
V_0_ (pixel)	0.7964
K_1_	0.0003
K_2_	0.0070

**Table 4:** Pose calculation uncertainty.

Pose Calculation Parameters	Uncertainty
X (mm)	0.0234
Y (mm)	0.0255
Z (mm)	0.0441
A (rad)	0.0000991
B (rad)	0.0000930
C (rad)	0.0000030

**Table 5:** Hand–eye calibration uncertainty.

Hand–Eye Calibration Parameters	Uncertainty
X (mm)	1.9405
Y (mm)	1.4926
Z (mm)	1.5162
A (rad)	0.0015775
B (rad)	0.0017408
C (rad)	0.0020956

**Table 6:** Assembly pose uncertainty.

Assembly Pose Parameters	Uncertainty
X (mm)	2.5023
Y (mm)	2.0291
Z (mm)	1.5122
A (rad)	0.0015411
B (rad)	0.0017511
C (rad)	0.0020976

**Table 7:** Mechanical parameters of the parallel robot.

Parameter	Value
Number of positioners	3
Number of total axes	9
Load capacity	0.5 t
Moving speed	0–0.5 m/min
Maximum acceleration	0.1 m/s^2^
Displacement resolution	0.001 mm
X stroke	±50 mm
Y stroke	±50 mm
Z stroke	150 mm
A(RX) stroke	±14°
B(RY) stroke	±7°
C(RZ) stroke	±5°
Positioning accuracy	≤0.01 mm
Repeated positioning accuracy	≤0.005 mm

**Table 8:** Experiment parameters set for dropout rate control experiments.

Network	Dropout Rate
LeNet*	-
Control group 1	0.2
Control group 2	0.3
Control group 3	0.5

**Table 9:** Experiment parameters set for L2 regularization parameter control experiments.

Network	L2 Regularization Parameter
LeNet*	-
Control group 1	0.0005
Control group 2	0.001
Control group 3	0.002

**Table 10:** Experiment parameters set for the network layer control experiments.

Network	Number of Convolutional Layers
Original network	2
Control group 1	4
Control group 2	6
Control group 3	10

**Table 11:** Experiment parameters set for the pooling layer control experiments.

Network	Number of Pooling Layers
Original network	2, preserve the complete pooling layers
Control group 1	1, preserve the pooling layer before the linear layer
Control group 2	0

**Table 12:** Experiment parameters set for the convolution kernel size control experiments.

Network	Convolution Kernel Size
Original network	3
Control group 1	1
Control group 2	5

**Table 13:** Experiment parameters set for the activation function control experiments.

Network	Activation Function
Original network	ReLU
Control group 1	LeakyReLU
Control group 2	Tanh
Control group 3	Sigmoid

**Table 14:** Experiment parameters set for the normalization technique control experiments.

Network	Normalization Technique
Original network	-
Control group 1	BatchNorm1d after convolution layer
Control group 2	BatchNorm1d after convolution layer and fully connected layer
Control group 3	BatchNorm1d after convolution layer and LayerNorm after fully connected layer

**Table 15:** Experiment parameters set for the pooling layer control experiments.

Network	Layer 1 Parameters	Layer 2 Parameters
Original network	Number of input channels: 1 Number of output channels: 6	Number of input channels: 6 Number of output channels: 16
Control group 1	Number of input channels: 1 Number of output channels: 16	Number of input channels: 16 Number of output channels: 32
Control group 2	Number of input channels: 1 Number of output channels: 64	Number of input channels: 64 Number of output channels: 128
Control group 3	Number of input channels: 1 Number of output channels: 128	Number of input channels: 128 Number of output channels: 512

**Table 16:** Experiment parameters set for the learning rate control experiments.

Network	Learning Rate
Original network	0.001
Control group 1	0.005
Control group 2	0.0005
Control group 3	0.0001

**Table 17:** Experiment parameters set for the epoch control experiments.

Network	Epoch
Original network	300
Control group 1	600
Control group 2	100

**Table 18:** Optimal hyperparameters of OECNN.

Hyperparameter	Set
Convolutional layer 1	Number of input channels: 1
	Number of output channels: 6
Convolutional layer 2	Number of input channels: 6
	Number of output channels: 16
Learning rate	0.001
Epoch	100

**Table 19:** Average and standard deviation of positioner drive volume estimation errors.

No.	Method	Average (mm)	Standard Deviation (mm)
1	Tsai	0.9104	0.2062
2	Direct	0.1786	0.0409
3	PECNN	0.1139	0.0224
4	Proposed OECNN	0.0103	0.0016

**Table 20:** Pose uncertainty before optimization.

Parameters	Pose Calculation Uncertainty	Hand–Eye Calibration Uncertainty	Assembly Pose Uncertainty
X (mm)	0.0472	2.0122	2.9694
Y (mm)	0.0504	1.8765	3.0166
Z (mm)	0.0633	1.5543	1.5835
A (rad)	0.0001344	0.0027065	0.0023345
B (rad)	0.0001207	0.0023488	0.0023883
C (rad)	0.0000065	0.0022067	0.0022261

**Table 21:** Final assembly uncertainty.

Parameters	Final Assembly Uncertainty
X (mm)	0.0466
Y (mm)	0.0491
Z (mm)	0.0472
A (rad)	0.0000346
B (rad)	0.0000321
C (rad)	0.0000057

## Data Availability

The raw data supporting the conclusions of this article will be made available by the authors upon request.
